# Spider surgical system versus multiport laparoscopic surgery: performance comparison on a surgical simulator

**DOI:** 10.1186/s12893-015-0038-9

**Published:** 2015-05-03

**Authors:** Domenico Giannotti, Giovanni Casella, Gregorio Patrizi, Giorgio Di Rocco, Lidia Castagneto-Gissey, Alessio Metere, Maria Giulia Bernieri, Anna Rita Vestri, Adriano Redler

**Affiliations:** 1 Department of Surgical Sciences Policlinico “Umberto I, “Sapienza” University of Rome, Viale Regina Elena, 324 Rome, Italy; 2 Department of Radiology, Oncology and Pathology, “Sapienza” University of Rome, Rome, Italy; 3 Department of Public Health and Infectious Diseases, “Sapienza” - University of Rome, Rome, Italy

**Keywords:** Surgical simulators, Surgical training, Spider, Medical education, Sils, Laparoscopy

## Abstract

**Background:**

The rising interest towards minimally invasive surgery has led to the introduction of laparo-endoscopic single site (LESS) surgery as the natural evolution of conventional multiport laparoscopy. However, this new surgical approach is hampered with peculiar technical difficulties. The SPIDER surgical system has been developed in the attempt to overcome some of these challenges. Our study aimed to compare standard laparoscopy and SPIDER technical performance on a surgical simulator, using standardized tasks from the Fundamentals of Laparoscopic Surgery (FLS).

**Methods:**

Twenty participants were divided into two groups based on their surgical laparoscopic experience: 10 PGY1 residents were included in the inexperienced group and 10 laparoscopists in the experienced group. Participants performed the FLS pegboard transfers task and pattern cutting task on a laparoscopic box trainer. Objective task scores and subjective questionnaire rating scales were used to compare conventional laparoscopy and SPIDER surgical system.

**Results:**

Both groups performed significantly better in the FLS scores on the standard laparoscopic simulator compared to the SPIDER.

Inexperienced group: Task 1 scores (median 252.5 vs. 228.5; p = 0.007); Task 2 scores (median 270.5 vs. 219.0; p = 0.005).

Experienced group: Task 1 scores (median 411.5 vs. 309.5; p = 0.005); Task 2 scores (median 418.0 vs. 331.5; p = 0.007).

Same aspects were highlighted for the subjective evaluations, except for the inexperienced surgeons who found both devices equivalent in terms of ease of use only in the peg transfer task.

**Conclusions:**

Even though the SPIDER is an innovative and promising device, our study proved that it is more challenging than conventional laparoscopy in a population with different degrees of surgical experience. We presume that a possible way to overcome such challenges could be the development of tailored training programs through simulation methods. This may represent an effective way to deliver training, achieve mastery and skills and prepare surgeons for their future clinical experience.

## Background

Since Navarra performed the first single incision laparoscopic cholecystectomy (SILC) in 1997 [1], the rapid advances in minimally invasive surgery have led to the development of several single-port laparoscopic techniques and instruments. The variety of devices and trademarks have spawned a true “battle of acronyms” (SILS, SSLS, SPA, SSL, OPUS, TUES, E-NOTES, NOTUS, etc.), without a definitive consensus name for this new technique of minimally invasive surgery [2].

In 2008 the NOTES Working Group of the Endourological Society and the Laparoendoscopic Single-Site Surgery Consortium for Assessment and Research tried to standardize the terminology to LESS (Laparoendoscopic single site surgery). LESS was defined as any minimally invasive surgical procedure, performed through a single incision/location, using conventional laparoscopic or newly emerging instrumentations. Although the feasibility of LESS has been demonstrated in general, gynecologic, urologic and bariatric surgery, several limitations still affect the single site approach such as lack of instrument triangulation, in-line viewing, cross-handed instrumentation and intra- and extra-corporeal instrument collisions [2]. In the past 5 years several attempts have been made to overcome these drawbacks with the introduction of pre-bent rigid, flexible and articulating laparoscopic instruments. Covidien, Inc. (Norwalk, CT, USA), Novare RealHand HD (Novare Surgical Systems, Cupertino, CA) and Cambridge Endo (Cambridge Endoscopic Devices, Framingham, MA) instruments articulate into the abdomen with different degrees of freedom providing better triangulation. Nevertheless definitive solutions were not provided to avoid cross-handed instrumentations and collisions. Robotic technology is being proposed to reduce the technical challenges of LESS [3]. The da Vinci Single-Site surgical system (Intuitive Surgical, Sunnyvale, CA, USA), the first commercial robot with a kit designed for LESS, allows to overcome the constraint of cross-handed operation by switching the right and left instruments and enables the surgeon’s hand to control the instrument on the same side of the screen [4,5]. As interest in this new technology continues to grow, a more and more flexible continuum (e.g. IREP robot [6], i-Snake robot [7]) and modular designed robots (e.g. SPRINT robot [8]) are going to be introduced in the surgical market, but outcomes and costs still remain to be defined.

In this context, the research of an ideal single-site surgical platform that might replicate multiport laparoscopy is far from over. In 2009, TransEnterix (Durham, NC, USA) introduced the SPIDER (Single Port Instrument Delivery Extended Reach) surgical system, a single incision surgical device with multiple working channels for rigid and flexible instruments. The system opens up with an umbrella-like system within the abdomen restoring the concept of triangulation without cross-handed instrumentation.

Although the SPIDER has been applied to a variety of surgical procedures [9], there are very few studies evaluating this new surgical platform. Our study aimed to compare standard laparoscopy and SPIDER technical performance on a surgical simulator, using standardized tasks from the Fundamentals of Laparoscopic Surgery (FLS).

## Methods

The study was performed in the Department of Surgical Sciences at “Sapienza”—University of Rome, Rome, Italy. The study was approved by the local Ethics Committee (protocol 518/13) which is the Ethical Committee of the Umberto I Hospital, “Sapienza”-University of Rome. All subjects were enrolled into the study on a voluntary basis and each participant provided full written informed consent. All participants completed a questionnaire assessing demographics as well as number and type of previous laparoscopic procedures. We recruited 20 participants and divided them into two groups according to their experience in laparoscopic surgery. The first group, the inexperienced group, included 10 post-graduate first year residents (PGY 1) in general surgery (mean age 26.1 ± 1.9 years) with none or low laparoscopic experience (less than 5 laparoscopic procedures, all of them as camera operator). The second group, the LAP experienced group, included 10 surgeons (mean age 36.2 ± 3.6 years), performing more than 50 laparoscopic procedures as first operator.

All subjects had no prior experience with FLS and LESS surgery.

Participants performed the FLS pegboard transfer task and the FLS pattern cutting task on a laparoscopic box trainer, using at first conventional laparoscopy for familiarization, then the SPIDER surgical system. To avoid potential outcome inhomogeneity, every participant was blinded to other surgeons’ performances.

### FLS tasks

Fundamentals of Laparoscopic Surgery (FLS) is a program of SAGES and the American College of Surgeons (SAGES/ACS, FLS Program, Los Angeles, CA, USA) designed to teach and evaluate the fundamental skills for laparoscopic surgery. The manual skills component is based on the McGill Inanimate System for Training and Evaluation of Laparoscopic Skills (MISTELS) program which consists of five basic tasks performed on a laparoscopic box trainer: pegboard transfer, pattern cutting, endo-loop placement, intracorporeal and extracorporeal knot [10]. In order to evaluate the performance differences between SPIDER and conventional laparoscopy, we chose **t**he pegboard transfer and the pattern cutting task. These tasks are not technically challenging and suitable even for beginners. The pegboard transfer task (Task 1) requires the operator to grasp six small pegs from a board, transfer them to the other hand, and place them on a second pegboard. The procedure is then reversed.

As the study included novice participants, the cutoff time was increased from 300 to 600 seconds.

According to MISTELS, the penalty score was defined as the percentage of pegs not transferred as a result of being dropped outside the field of view.

The pattern cutting task (Task 2) requires the participant to cut out a predrawn circle 5 cm in diameter from a 10 · 10 cm^2^ piece of gauze suspended between clips. One hand should be used to provide traction on the gauze using the grasper and to place the gauze at more favorable angles for the cutting hand.

As in the first task, the cutoff time was set at 600 seconds. According to MISTELS, the penalty was calculated as the percentage of the area of deviation from a perfect circle.

### LAP simulator

The system used in the study was the Simulab LapTrainer (Simulab, Seattle, Washington). It consists of a 22″ h, 18″ w, 8″ d modular plastic box, a boom mounted, fully adjustable 1080p high definition camera with auto-focus, and a Universal Serial Bus (USB) 2.0 card connected to a 17″ laptop monitor. Standard-length laparoscopic instruments were inserted through two 12-mm working ports positioned approximately 18 cm apart in the pliable cover.

### SPIDER simulator

The Simulab LapTrainer was modified to perform FLS tasks using the SPIDER surgical system.

A single 1.8 cm opening was made on the pliable cover of the box trainer, positioned between the previous port positions. The SPIDER surgical system was inserted through the single opening and was stabilized with an external support arm device.

To perform the FLS task we used a second-generation SPIDER surgical system with a vertebral design of the instrument delivery tubes.

### Testing procedure and score calculation

**Figure 1:**
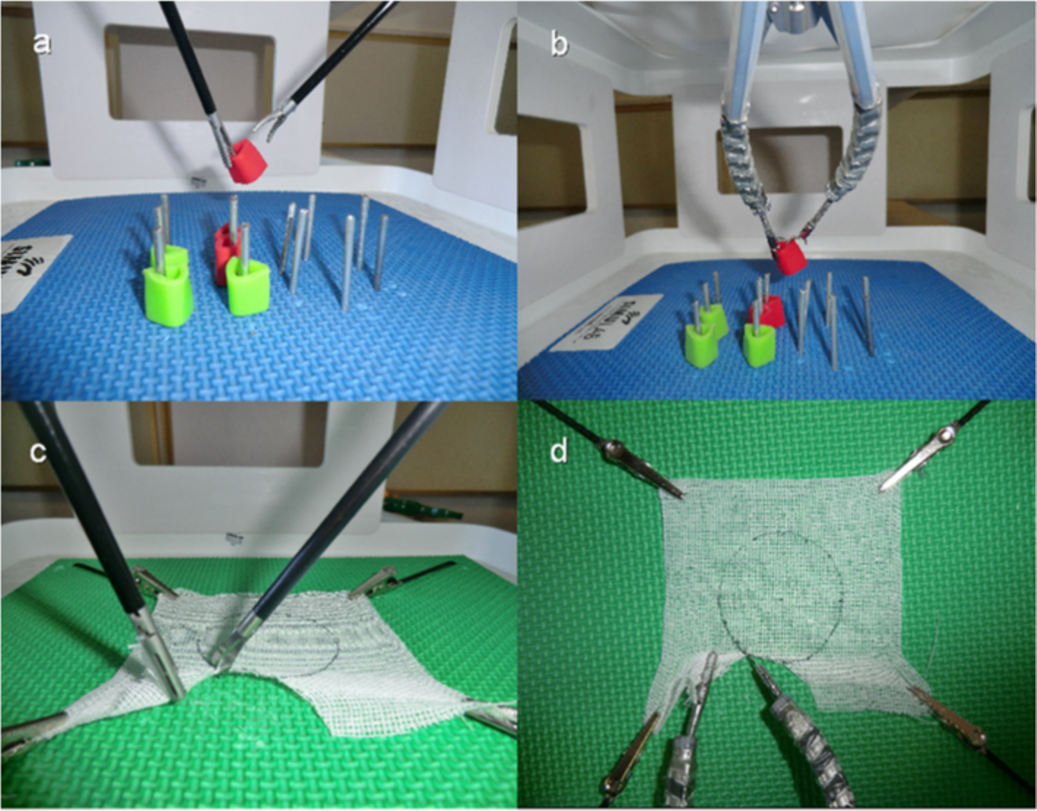
Fundamentals of Laparoscopic Surgery: Conventional laparoscopy versus SPIDER surgical system. **a)** Peg transfer task on multiport laparoscopic platform; **b)** Peg transfer task on SPIDER platform; **c)** pattern cutting task on multiport laparoscopic platform; **d)** pattern cutting task on SPIDER platform.

Before performing tasks, all participants viewed a FLS instructional video illustrating the ideal way to perform each exercise.Testing sessions were conducted on two consecutive days. On the first day, both groups sequentially performed the pegboard transfer (Figure 1a) and the pattern cutting task (Figure 1c) using the LAP simulator. On the second day the same tasks were repeated using the SPIDER simulator (Figure 1b,1d).

Objective task score and subjective questionnaire rating scales were used to compare conventional laparoscopy and the SPIDER surgical system.

According to established methods of score calculation [8], each task was scored using the following formula in which higher scores reflect better performances:

$$ \mathrm{Task}\ \mathrm{score} = \mathrm{cutoff}\ \mathrm{time}\ \hbox{--}\ \mathrm{completion}\ \mathrm{time}\ \hbox{--}\ \mathrm{penalty}\ \mathrm{score} $$

Time and penalty measurements were performed by a proctor experienced in FLS evaluation.

A questionnaire was administered to all participants after each task to define conventional laparoscopy and SPIDER’s ease of use.

The subjective questionnaire rating was based on a six points Likert scale (1: very difficult, 2: difficult, 3: somewhat difficult, 4: somewhat easy, 5: easy, 6: very easy).

### Statistical analysis

Continuous variables are presented as mean ± standard deviation. We assessed the normality of data with the Shapiro–Wilk test. Data of task score and subjective questionnaire ratings do not follow a normal distribution and therefore were reported as median and interquartile range. To evaluate the differences within groups we perform Wilcoxon test. To compare the performance of a specific group on LAP simulator versus SPIDER, we used the Mann–Whitney test and the Median test. A probability value of less than 0.05 was considered as statistically significant. All analyses were carried out with STATA v.12.

## Results

### Task score

Comparing multiport laparoscopy and SPIDER surgical system in the inexperienced group we found significant differences in Task 1 scores (median 252.5 vs. 228.5; p = 0.007 ) and in Task 2 scores (median 270.5 vs. 219.0; p = 0.005).

Also the experienced group performed significantly better in Task 1 scores (median 411.5 vs. 309.5; p = 0.005 ) and in Task 2 scores (median 418.0 vs. 331.5; p = 0.007) on the Lap simulator.

When comparing LAP scores between groups, the experienced group had significantly higher scores in both Task 1 (median 411.5 vs. 252.5; p = 0.002 ) and Task 2 (median 418.0 vs. 270.5; p = 0.005). Likewise, when examining SPIDER scores, the experienced group performed significantly better in both tasks (median 309.5 vs. 228.5; p = 0.012 ), (median 331.5 vs. 219.0; p = 0.01).

The results of Tasks 1 and 2 are summarized in Tables 1 and 2 respectively.

**Table 1:** **Task 1 (Peg transfer) FLS scores and subjective evaluation of conventional laparoscopy (LAP) vs. SPIDER**

Inexperienced Group					
	LAP		SPIDER		
	Median	IQR	Median	IQR	P-value
**FLS score**	252.5	227.7-347.5	228.5	206.0-270.5	0.007
**Ease of use**	5.0	3.0-4.0	4.0	2.7-3.0	0.093
**LAP experienced Group**					
	**LAP**		**SPIDER**		
	**Median**	**IQR**	**Median**	**IQR**	**P-value**
**FLS score**	411.5	399.2-421.2	309.5	290.7-338.5	0.005
**Ease of use**	5.0	5.0-5.2	3.0	3.0-4.0	0.004

**IQR:** interquartile range.

**Table 2:** **Task 2 (Pattern cutting) FLS scores and subjective evaluation using conventional laparoscopy (LAP) and SPIDER**

Inexperienced Group					
	LAP		SPIDER		
	Median	IQR	Median	IQR	P-value
**FLS score**	270.5	242.2-347.7	219.0	183.7.-252.5	0.005
**Ease of use**	3.0	2.7-4.0	2.0	2.0-3.0	0.015
**LAP experienced Group**					
	**LAP**		**SPIDER**		
	**Median**	**IQR**	**Median**	**IQR**	**P-value**
**FLS score**	418.0	352.5-432.2	331.5	233.0-359.2	0.007
**Ease of use**	5.0	5.0-5.2	3.0	3.0-3.2	0.002

**IQR:** interquartile range.

### Questionnaire rating scales

Among the inexperienced group, the ease of use of conventional laparoscopy and SPIDER surgical system did not differ in Task 1 (median 3 vs. 3; p = 0.093). Task 2 proved to be simpler with conventional laparoscopic instruments as opposed to the SPIDER system (median 3 vs. 2; p = 0.015).

Furthermore, experienced laparoscopic surgeons found it easier to execute both tasks through conventional laparoscopy rather than with the SPIDER system (Task 1: median 5 vs. 3 p = 0.004. Task 2: median 5 vs. 3 p = 0.002).

Comparing overall personal responses to the questionnaire between groups, on the laparoscopic simulator the experienced group showed significantly higher scores in both Task 1 (median 5 vs. 3 p = 0.002 ) and Task 2 (median 5 vs. 3 p = 0.0015). On the SPIDER simulator no significant differences were found between groups in Task 1 (median 3 vs. 3 p = 0.648 ) whereas significant differences were seen in Task 2 (median 3 vs. 2 p = 0.0113).

The results of Tasks 1 and 2 are summarized in Tables 1 and 2 respectively.

## Discussion

A progressive interest towards minimally invasive procedures has grown in the latest decades leading to the recent introduction of LESS surgery which represents an evolutionary change compared to the classical laparoscopic approach. However, such surgical innovation is associated with peculiar and unique challenges in addition to those already given by conventional laparoscopy [2]. Some of the disadvantages are represented by the lack of triangulation, in-line viewing, cross-handed operating, instrument collisions, crowding of the instrument handles and the ability to target only one or two abdominal quadrants. These ergonomic limitations may transform even simple maneuvers in complicated ones. Even though no ideal instrument still exists, new technologies aim to find a solution to the obstacles preserving its advantages. Articulating instruments can partially restore triangulation also decreasing collisions and in-line viewing.

Although these systems provide better triangulation, the problem of cross-handed instrumentation is yet to be solved. Indeed, without crossing instruments, the surgeons’ hands operate in a small field and tend to collide despite the degrees of freedom of the instruments’ tips.

The SPIDER surgical system represents an innovative device, introduced in 2009, with the purpose of overcoming several of the limitations mentioned above. It is a disposable single-incision device provided with ergonomic arms and multiple working channels for rigid and flexible instruments. Some of its advantages include the absence of instrument collisions, additional degrees of freedom and an appropriate operative exposure allowing the possibility of triangulation.

Although the SPIDER surgical system has been successfully applied to several surgical procedures including cholecystectomy, nephrectomy and colectomy [9], most studies are referred to case reports or animal models [11-14].

The only human series evaluating the SPIDER platform was retrospectively collected by Gonzalez et al. [15]. These authors compared their first single-incision cholecystectomies performed by standard laparoscopic, robotic and SPIDER platforms with similar results for most of the parameters considered. However, certain selection biases due to differences in mean age and body mass index of patients were identified among the three groups.

Laparoscopic simulators could represent a safe and objective tool for assessing not only surgeon’s technical skills [16-22] but also novel surgical instruments [23-25]. Indeed, they can reduce the impact of biases associated to patients’ variability by providing a perfectly reproducible and measurable evaluation of the instrument’s validity.

Our study evaluated the performance of residents and surgeons with different degrees of experience facing two FLS standardized tasks on a laparoscopic box trainer, using conventional laparoscopy and the SPIDER surgical system. Moreover, questionnaire rating scales were administered to evaluate the ease of use of the surgical devices from a personal point of view.

Overall objective task scores showed that the SPIDER surgical device was more challenging than conventional laparoscopy. As expected, the experienced group performed globally better than the inexperienced one in terms of FLS score on both conventional laparoscopy and SPIDER simulator platforms.

**Figure 2:**
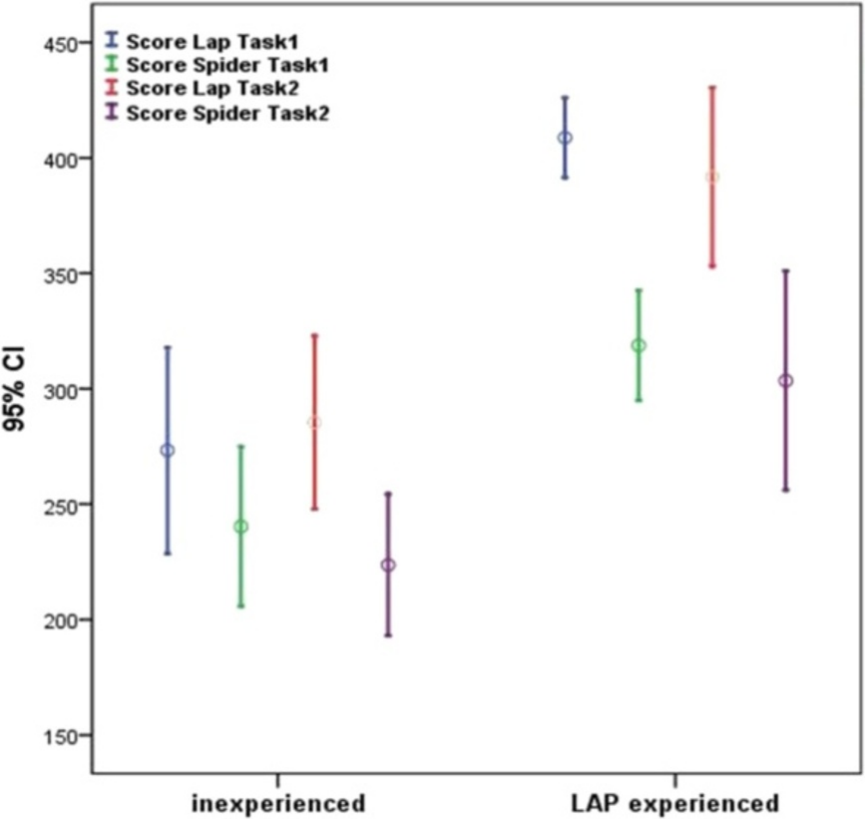
Task 1 and task 2 FLS scores obtained using conventional laparoscopy ( Score Lap) and SPIDER surgical system (Score SPIDER).

Among the experienced group, significant differences were found between conventional laparoscopy and SPIDER surgical system in Task 1 scores (median 252.5 vs. 228.5; p = 0.007 ) and Task 2 scores (median 270.5 vs. 219.0; p = 0.005 ). Also in the inexperienced group we found significant differences in Task 1 scores (median 252.5 vs. 228.5; p = 0.007 ) and Task 2 scores (median 270.5 vs. 219.0; p = 0.005 ), but the gap in task 1 median scores (median 252.5 vs. 228.5) was smaller compared to the experienced group (median 411.5 vs. 309.5) (Figure 2).

**Figure 3:**
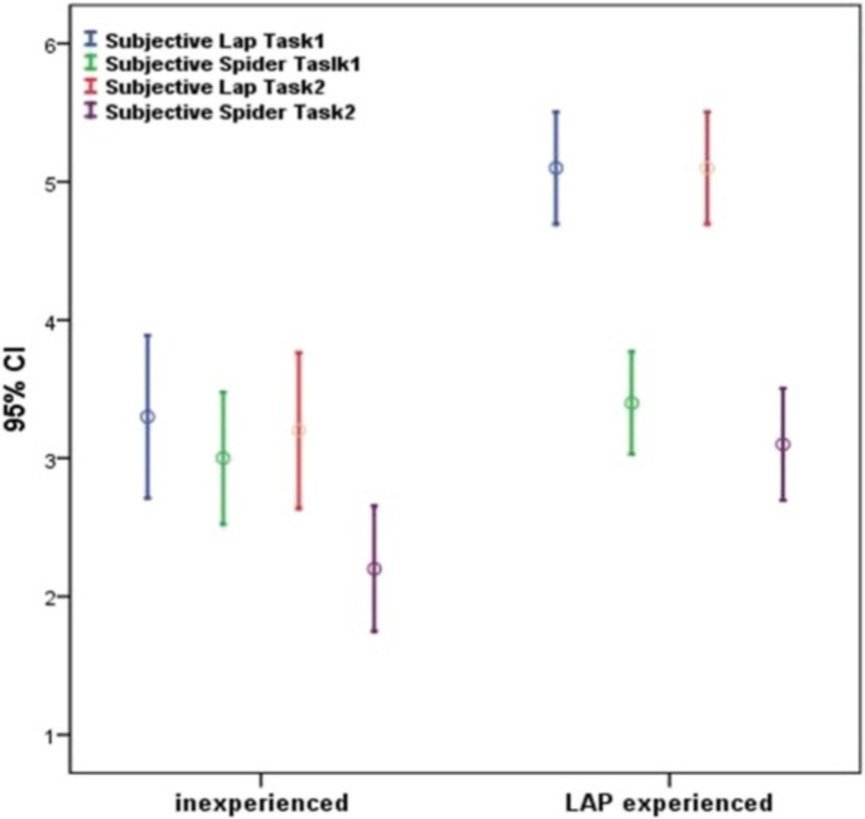
Ease of use of conventional laparoscopy ( Subjective Lap) and SPIDER surgical system (Subjective SPIDER) in task 1 and task 2.

Furthermore, among the inexperienced group, the ease of use of conventional laparoscopy and SPIDER surgical system did not differ in Task 1 (median 3 vs. 3; p = 0.093) while experienced surgeons found it easier to execute both tasks through conventional laparoscopy rather than with the SPIDER system (Task 1: median 5 vs. 3 p = 0.004. Task 2: median 5 vs. 3 p = 0.002) (Figure 3).

A possible explanation is that residents were inexperienced with both multiport laparoscopy and SPIDER surgical system while the experienced surgeons were familiar only with multiport laparoscopy. In fact, Lewis et al. demonstrated that previous laparoscopic experience influences the ability to perform simulated tasks with single-incision laparoscopic devices [26].

On the other hand, in Task 2 the SPIDER was more challenging for both groups with significantly lower FLS scores and subjective questionnaire ratings.

This is probably due to the fact that this task requires appropriate traction, exposure of the surgical field and a greater precision when cutting the gauze. Even though the second generation SPIDER is manufactured to increase maneuverability and precision of movement, thanks to the new vertebral design, there is still an undesirable elastic recoil.

Indeed, we were able to ascertain using the SPIDER simulator that it is challenging to gauge with precision the amount of traction and therefore the exposure of the cutting line. At the same time the excessive traction needed has sometimes caused the loss of the instrument’s grip with consequent prolonged task accomplishment time.

Furthermore, the SPIDER system provides a lower range of motion compared to conventional laparoscopy especially at the boundaries of the operative field. This problem can be overcome by time-demanding external maneuvers on the supporting arm of the SPIDER itself.

Our study should be regarded in the context of some limitations. First of all, it might be interesting to perform further studies in order to investigate if a more organized and structured training with both platforms could somehow modify the results. Indeed, the number of reiterations needed to achieve an objective evaluation is strictly dependent on individual experience and therefore extremely hard to define. We already faced this problem in previous studies and, once again, we believe the best option is to perform a single test session to avoid bias [27]. Another limitation was the rather small number of tasks evaluated. We used only two FLS tasks since our aim was to check the performances of inexperienced operators avoiding therefore more challenging tasks as knot tying. Furthermore, since inclusion criteria were no previous experience of FLS and LESS, we reached only ten experienced surgeons who met both criteria. Based on such limitation, we then decided to compare them only with ten novices. This basically represents a convenience sample. Finally, we are planning to perform dedicated studies in the near future in order to compare the SPIDER with other single incision devices on a greater number of participants.

## Conclusions

Fundamental differences exist between multiple-port laparoscopy and single-site incision strategies.

The main advantages of LESS are those of reducing postoperative pain and of improving cosmetics, nonetheless it is also burdened with a number of disadvantages in addition to those already encountered in traditional laparoscopy [2]. A possible way to overcome some of these limitations, unfamiliarity with the instrumentation being one of them, could be the development of tailored training programs through simulation methods. This could possibly represent an effective mean to deliver training, achieve mastery and skills and prepare surgeons for their future clinical experience.
